# Comprehensive genetic variant analysis reveals combination of *KRAS* and *LRP1B* as a predictive biomarker of response to immunotherapy in patients with non-small cell lung cancer

**DOI:** 10.1186/s13046-025-03342-6

**Published:** 2025-02-27

**Authors:** Ella A. Eklund, Johanna Svensson, Louise Stauber Näslund, Maria Yhr, Sama I. Sayin, Clotilde Wiel, Levent M. Akyürek, Per Torstensson, Volkan I. Sayin, Andreas Hallqvist, Sukanya Raghavan, Anna Rohlin

**Affiliations:** 1 Sahlgrenska Center for Cancer Research, Department of Surgery, Institute of Clinical Sciences, University of Gothenburg, Gothenburg, Sweden; 2 Wallenberg Centre for Molecular and Translational Medicine, University of Gothenburg, Gothenburg, Sweden; 3 Department of Oncology, Sahlgrenska University Hospital, Gothenburg, Sweden; 4 Department of Clinical Genetics and Genomics, Sahlgrenska University Hospital, Gothenburg, Sweden; 5 Department of Laboratory Medicine, Institute for Biomedicine, Sahlgrenska Academy, University of Gothenburg, Gothenburg, Sweden; 6 Department of Clinical Pathology, Department of Biomedical and Clinical Sciences, Linköping University, Linköping, Sweden; 7 Department of Clinical Pathology, Institute for Biomedicine, Sahlgrenska University Hospital, Gothenburg, Sweden; 8 Department of Pulmonary Medicine, Skaraborg Hospital, Skövde, Sweden; 9 Department of Oncology, Institute for Clinical Sciences, Sahlgrenska Academy, University of Gothenburg, Gothenburg, Sweden; 10 Department of Microbiology and Immunology, Sahlgrenska Center for Cancer Research, Institute for Biomedicine, University of Gothenburg, Gothenburg, Sweden; 11 Department of Clinical Immunology and Transfusion Medicine, Sahlgrenska University Hospital, Gothenburg, Sweden

**Keywords:** Variant classification, *LRP1B*, *KRAS*, *TP53*, ICB, NSCLC, Biomarker

## Abstract

**Background:**

In non-small cell lung cancer (NSCLC), the rapid advancement of predictive genetic testing of tumors by identifying specific pathogenic driver variants has significantly improved treatment guidance. However, immune checkpoint blockade (ICB) is typically administered to patients with tumors in the absence of such driver variants. Since only about 30% of patients will respond to ICB treatment, identifying novel genetic biomarkers of clinical response is crucial and will improve treatment decisions. This prospective clinical study aims to combine molecular biology, advanced bioinformatics and clinical data on response to treatment with ICB from a prospective cohort of NSCLC patients to identify single or combination of genetic variants in the tumor that can serve as predictive biomarkers of clinical response.

**Methods:**

In this prospective bi-center clinical study, we performed next-generation sequencing (NGS) of 597 cancer-associated genes in a prospective cohort of 49 patients as the final cohort analyzed, with stage III or IV NSCLC, followed by establishment of an in-house developed bioinformatics-based molecular classification method that integrates, interprets and evaluates data from multiple databases and variant prediction tools. Overall survival (OS) and progression-free survival (PFS) were analyzed for selected candidate genes and variants identified using our novel methodology including molecular tools, databases and clinical information.

**Results:**

Our novel molecular interpretation and classification method identified high impact variants in frequently altered genes *KRAS*, *LRP1B*, and *TP53*. Analysis of these genes as single predictive biomarkers in ICB-treated patients revealed that the presence of likely pathogenic variants and variants of unclear significance in *LRP1B* was associated with improved OS (*p* = 0.041). Importantly, further analysis of variant combinations in the tumor showed that co-occurrence of *KRAS* and *LRP1B* variants significantly improved OS (*p* = 0.003) and merged PFS (*p* = 0.008). Notably, the triple combination of variants in *KRAS*, *LRP1B*, and *TP53* positively impacted both OS (*p* = 0.026) and merged PFS (*p* = 0.003).

**Conclusions:**

This study suggests that combination of the *LRP1B* and *KRAS* variants identified through our novel molecular classification scheme leads to better outcomes following ICB treatment in NSCLC. The addition of *TP53* improves the outcome even further. To our knowledge, this is the first report indicating that harboring a combination of *KRAS*, *LRP1B*, and *TP53* variants can significantly enhance the response to ICB, suggesting a novel predictive biomarker combination for NSCLC patients.

**Supplementary Information:**

The online version contains supplementary material available at 10.1186/s13046-025-03342-6.

## Background

In the era of precision medicine, the identification of specific molecular variants in tumors as biomarkers of clinical response can guide individualized treatment options. In non-small cell lung cancer (NSCLC), there has been rapid development of targeted treatments and predictive genetic testing as tools to guide treatment through the identification of specific pathogenic driver variants (also defined as driver mutations). Currently, tumor tissue is analyzed mainly with next-generation sequencing (NGS) for activating pathogenic variants or rearranged fusion oncogenes in *EGFR*,* ALK*,* BRAF*,* MET* (exon 14 skipping), *KRAS*,* ROS1*, *RET* and *NTRK.* However, only approximately 50% of patients have a targetable pathogenic DNA variant [1], and for patients without access to targeted first line treatment including patients with pathogenic *KRAS* variants, chemotherapy, immune checkpoint blockade (ICB), combined chemotherapy and ICB or combined ICB are the main treatment options [2].

Currently, programmed death-ligand 1 (PD-L1) expression is the standard predictive biomarker for response to ICB therapy for NSCLC patients without targetable pathogenic DNA variants [3]. PD-L1 expression in tumor tissue is estimated via immunohistochemistry and for most indications evaluated as the tumor proportion score (TPS) [4]. Patients with high PD-L1 expression (≥ 50%) have a higher probability of responding to ICB monotherapy compared to patients with low (1–49%) or negative (< 1%) PD-L1 expression [5, 6]. However, the accuracy of PD-L1 expression as an individual prediction tool is debated since PD-L1-negative patients have been reported to be responders to ICB [7, 8]. Along these lines, a recent meta-analysis of five randomized controlled trials with monotherapy of pembrolizumab (PD-1 blockade) showed significantly improved overall survival (OS) compared to chemotherapy also for patients with PD-L1 TPS < 1% [9].

At present, patients with stage IV disease can be treated with ICB monotherapy if PD-L1 is expressed in ≥50% of tumor cells, whereas chemotherapy and ICB combinations and combined ICB can be administered regardless of PD-L1 expression [3, 4]. Since 2018, adjuvant ICB with curative intent after concurrent chemoradiotherapy has been available for patients with locoregional (stage III) PD-L1-positive disease [10], and recently, ICB has been introduced in treatment strategies including surgery with both neoadjuvant and adjuvant regimens [11, 12].

However, specific molecular markers for identifying patients who will respond to ICB are still lacking, as only 20-40% of patients currently benefit from ICB therapy [13–16]. Besides PD-L1 expression, there is only one biomarker for solid tumors that received FDA approval for treatment with pembrolizumab in the US in 2020 [17], defined as tumor mutational burden (TMB). TMB constitutes the number of somatic DNA variants in the tumor calculated per megabase (Mb) and  ≥10 mutations/Mb has been approved as a cutoff, associated with a favorable outcome after ICB treatment in some studies [18, 19]. The reliability of TMB, however, has been questioned and the indication has never been approved in the EU. Potential reasons include the lack of standardized laboratory methods for analyzing and calculating TMB, which has made it difficult to compare and find a relevant cutoff level regarding the number of variants that represent a high TMB. As a result, various cutoffs ranging from 10 to 20 variants/Mb have been suggested in additional studies [18–21]. There are also results reported contradictory to expected outcome, where patients with low TMB were responders and vice versa [20, 22, 23].

Regarding known tumor drivers in NSCLC and response to ICB, there are some data where tumors driven by *EGFR* variants and *ALK* rearrangement have been shown to be resistant to ICB regardless of PD-L1 expression status [24, 25]. *KRAS*, the most prevalent oncogenic driver in NSCLC, has been associated with a better response to ICB than patients not harboring variants in *KRAS*, but the findings have to date been inconclusive [26–31]. Single gene variants or multiple variants in different genes, referred to as co-mutations or co-variants, have been correlated with the response to ICB in different studies and the unclear correlation of the ICB response to *KRAS* pathogenic variants could be explained by co-variants in *STK11* and *KEAP1* that are associated with a poor response [32–35], whereas co-variants in *TP53* mostly are associated with a good response [36, 37]. Variants in the tumor suppressor *LRP1B* have recently been linked to better outcomes following ICB treatment in multiple cancers, including NSCLC [38, 39].

The use of broad NGS panels for screening of multiple genes in the diagnosis of NSCLC provides information about a large number of DNA variants that are not known drivers and there is a lack of solid evidence regarding their pathogenicity. The in-depth knowledge of how different variants in various genes cooperate based on their pathogenicity and how this relates to prediction of response is still in its early stages and presents large challenges. The molecular biomarkers identified to date have mainly been based on previously known pathogenic variants in well-known oncogenic drivers [40].

The classification of variants in less well-known genes as “pathogenic” or “benign” is often unclear and challenging because molecular tools and clinical information is lacking and not consistently evaluated according to guidelines such as the AMP and ACMG [40]. Currently, co-variants are not considered in clinical practice for selection of treatment options. Studies on the impact of co-variants on treatment response have reported results from retrospectively collected datasets with limited number of patients and not correlated with treatment regimen [41, 42]. Another molecular strategy for identifying new biomarkers is to study specific patterns of variants in tumors, known as mutational signatures [43–45].

In this prospective study, we analyzed variants in a well-characterized cohort of patients treated with ICB. We performed a genomic screening analysis of the tumors with a large NGS panel followed by a comprehensive variant analysis. The variant analysis included interpretation and classification of DNA variants, for which clinical evidence is lacking till date, using a large variety of databases and existing single prediction tools, with the aim to predict the pathogenicity of newly identified variants. Furthermore, we both analyzed the impact of selected single variants and combinations of variants on the clinical outcome of ICB treatment.

## Materials and methods

### Patient cohort

**Fig. 1:**
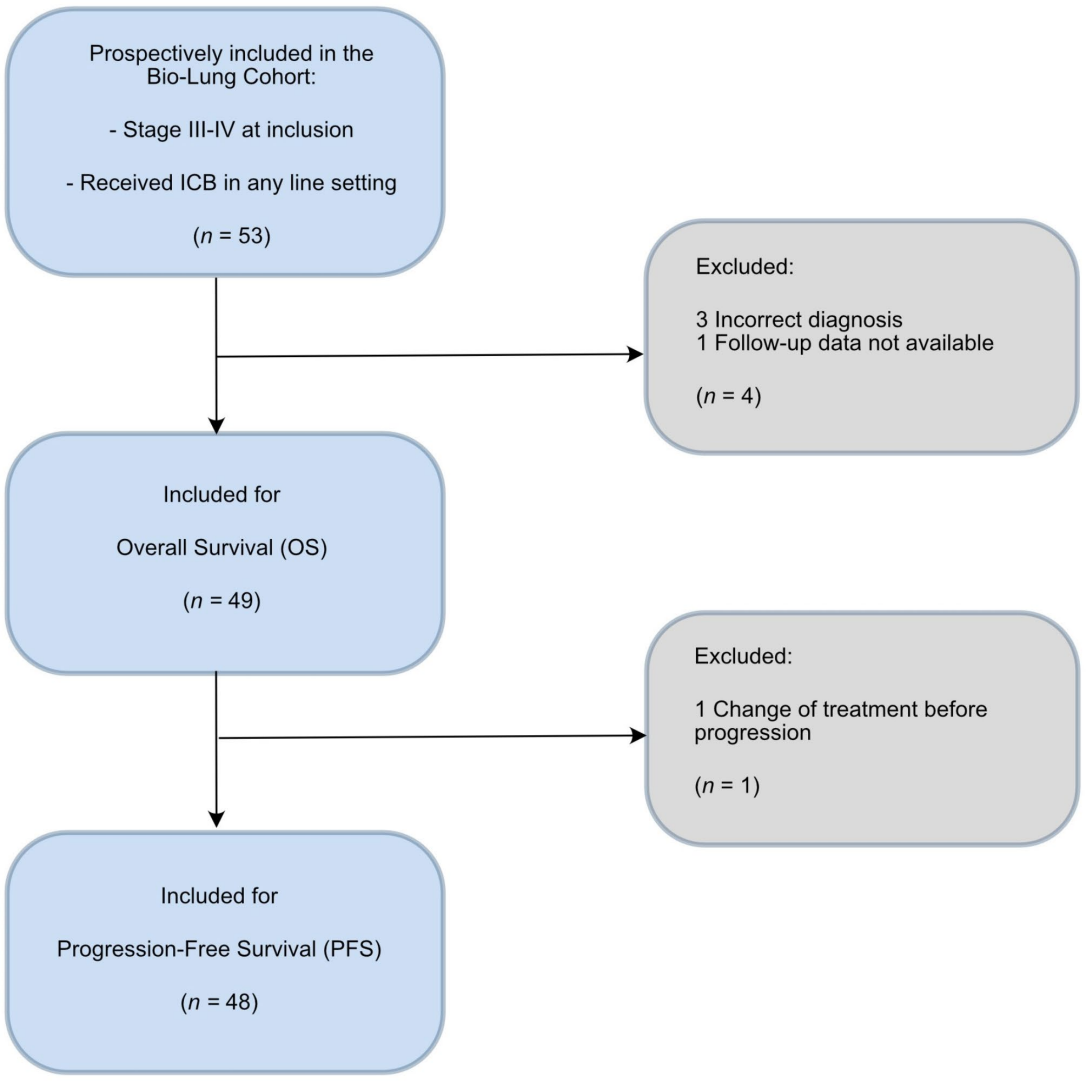
Patient inclusion and exclusion. Flow chart showing patients included in the analyses after applying exclusion criteria

We conducted a prospective bicentric study including 53 NSCLC patients (Bio-Lung cohort) with stage III or stage IV disease at inclusion, age ≥ 18 years, and receiving ICB in any line setting according to standard practice. Patients with treatment for newly diagnosed, recurrent disease and progressive disease were included. A single dose of ICB containing treatment was deemed to be sufficient. The patients were recruited between April 2019 and October 2021 and data cut of was 28th of February 2023. The study was approved by the Regional Ethics Review Board in Gothenburg, Sweden (Permit number 953/18), and all participating patients signed an informed consent. In the workup of the clinical data, two patients were excluded from further downstream analysis due to incorrect diagnoses, and another because of lack of follow-up data. In addition, through our mutational signaling analysis, we identified one patient with a skin primary tumor, and lung tumors were later considered to be metastatic lesions. This patient was therefore later excluded. As a result, 49 out of 53 patients were included in the final analysis (Fig. 1).

### Clinical response

The clinical response to treatment was determined based on the CT-scan results obtained every 3 months, in line with the immune-related Response Evaluation Criteria in Solid Tumors (irRECIST) algorithm, assessed by an oncologist according to clinical judgment. The clinical response was divided into complete response, partial response, stable disease, and progressive disease as previously described [46]. In this study, responders were defined as patients who did not have progressive disease at up to 9 months (3rd assessment) after the start of ICB therapy, confirming their clinical response to ICB.

### Isolation and sequencing of DNA from patient samples

Genomic DNA from FFPE tumor biopsies was initially analyzed in the clinical diagnosis workflow with the Oncomine™ Focus Assay (Thermo Fisher Scientific, Waltham, MA, USA), and these results are included in Additional file 1. The samples (*n =* 50) were then analyzed with a NGS panel, the INVIEW Oncoprofiling Panel (Oncopanel All in One v2.8, Eurofins Genomics (Europe Sequencing GmbH, Germany)), which included 597 cancer-associated genes (Additional file 2). All steps, including extraction of DNA from blood and FFPE samples, quantification, library preparation (Agilent Technologies, Santa Clara, CA, USA) and sequencing, were performed at Eurofins Genomics using in-house protocols and sequencing on the Illumina NovaSeq 6000 platform (Illumina, San Diego, CA, USA) with 2 × 150 bp paired-end reads and a limit of detection of 1% for insertions and deletions (indels) and single nucleotide variants (SNVs).

### Bioinformatic analysis and filtration of data

All data quality assessments, alignments and variant calling were performed at Eurofins Genomics (Europe Sequencing GMB, Germany) using the TNseq pipeline from Sentieon^®^ Genomics tools [47]. With the removal of duplicate reads, the average coverage for the FFPE samples was 775x, and for the corresponding blood samples, it was 1097x. High-quality reads with a Q-score above or equal to 30 were used in the downstream analysis. Paired germline and tumor analyses were performed to filter out germline DNA variants. Somatic-specific DNA variants in VCF files, including SNVs and indels, were further filtered in house using Alissa interpret (Agilent Technologies, Santa Clara, CA, USA) with a cutoff for population filtration of 1%. The data was not analyzed for copy number variants, structural variants, or fusion genes.

### Interpretation and classification of somatic DNA variants

All variants interpreted were manually reviewed in Integrative Genomics Viewer (IGV) for exclusion of technical artifacts [48]. A 5% variant allele frequency (VAF) cutoff was used, except for tumors with a low tumor-to-normal tissue ratio where a 3% cutoff was applied. Exonic variants, including nonsynonymous variants, splice variants (+/-2 bp), indels and known pathogenic synonymous variants, were analyzed. Exceptions were made for intronic variants in *BRCA1*,* BRCA2* and *MET* (exon 14), where intronic variants were also detected. Technically complex variants that were present in more than 10% of samples (except for recurrent known likely pathogenic and pathogenic variants), were excluded due to method- or panel-specific sequencing errors. DNA variants with a VAF < 0.1% according to the normal population database gnomAD (v2.1 and v3.1; https://gnomad.broadinstitute.org/) were further interpreted. Databases used included (OncoKB (https://www.oncokb.org/) and ClinVar (https://www.ncbi.nlm.nih.gov/clinvar/).

All DNA variants were classified into five interpretation categories, namely, pathogenic (P), likely pathogenic (LP), variant of unclear significance (VUS), likely benign and benign variants, where only the first three classes are presented. VUSs were classified based on several manually investigated molecular prediction criteria and databases, analyzed via an in-house developed point-based scoring scheme, and subclassed into four different classification categories (VUS-, VUS, VUS + and VUS++). The variants that had undergone the scoring system for classification (Additional file 3) were checked within AlphaMissense (assessed via MobiDetails), which in most cases agreed with the classification from the variant classification system. Loss of function variants in oncogenes were directly classified as VUS and were not further evaluated. Variants interpreted with scores of 3.5 or above based on information in several databases and prediction tools were classified as VUS + + and in the range of 2.5 to 3 as VUS+ (Additional file 3).

In total, 75 variants were classified as VUS- if they had a score below zero and were not included in further analyses. These variants were not present in a known driver gene (CancerGeneCensus (COSMIC v96), cancer-genes.org (Memorial Sloan Kettering Cancer Center, New York Cite, USA; q-value/FDR < 0.25), IntOgen) or a driver variant (CancerGenomeInterpreter) and were also classified as likely benign in VarSome.

### Databases used and exceptions from standard workflow for variant classification

For variants in *TP53*, *BRCA1* and *BRCA2*, additional locus-specific databases were used, including the *TP53* (https://tp53.isb-cgc.org) and Seshat (http://vps338341.ovh.net/) databases and the *BRCA1* and *BRCA2* databases (InterVar (https://wintervar.wglab.org/) and *BRCA* Exchange (https://brcaexchange.org/)). Variants in *POLE* and *POLD1* were classified as VUS when the variant was outside of the exonuclease domains, and no further information regarding pathogenicity was found. If the variant was identified in the exonuclease domain of the protein [49], it was included in the evaluation for classification.

### Mutational signature analysis

High-quality BAM files from tumors and blood from each patient sample were used as input files for the extraction of both intronic and exonic somatic variants with Mutect2 (GATK, v.4.1.3.0, Broad Institute of MIT and Harvard, Cambridge, MA, USA). FilterMutectCalls was applied for filtering with the default settings. Furthermore, the samples were grouped and analyzed in SigProfilerExtractor with filtered data from FFPEsig [50, 51]. Mutational signature analysis for single-base substitution was performed on all 50 patient samples, which were assigned to reference mutational signatures (v 3.3; COSMIC v96). In this analysis one patient was found to have a signature related to UV-light exposure that identified the origin of the primary tumor from the skin (Additional file 4). This patient was excluded from further analysis.

### TMB analysis

TMB was calculated by Eurofins Genomics by dividing the number of variants by the size of the targeted coding region in Mb. Only missense variants were included in the calculation. The calculation was performed using the following exclusion criteria: noncoding variants, variants listed as known somatic pathogenetic variants (COSMIC v71), known germline variants (dbSNP), variants with depth below 50x and allele frequency below 0.05, germline variants with more than two counts in gnomAD, and variants in tumor suppressor genes [52, 53].

### PD-L1 expression

PD-L1 expression was determined based on the percentage of tumor cells with positive membranous staining and was reported as the tumor proportion score (TPS): PD-L1-negative TPS < 1%, low TPS 1-49%, and high TPS ≥ 50%. PD-L1 expression was assessed using PD-L1 IHC 28 − 8 pharmDx (Agilent Technologies, Santa Clara, CA, USA) during routine diagnostic workup, and staining was analyzed by lung pathologists.

### cBioPortal data

To extend the dataset, we extracted data from cBioPortal, where progression-free survival (PFS) data and mutational status data were available for *KRAS*,* TP53*, and *LRP1B* including studies where immunotherapy was used as a first- or second-line treatment. For studies including *KRAS* and *TP53*, we found 331 patients [54–58], whereas for *LRP1B* we found 91 patients [54, 55]. These data were merged with our dataset, referred to here as merged PFS. In total, 379 patient samples were included in the merged PFS for *KRAS* and *TP53*. For *LRP1B*, a total of 139 patient samples were included in the merged PFS cohort.

### Statistical analysis

Clinical characteristics were summarized using descriptive statistics and evaluated with univariate analysis. Kaplan‒Meier survival curves were generated to assess overall survival (OS) and progression free survival (PFS). OS was defined as the time interval from the date of administration of first ICB containing treatment until death from any cause. PFS was defined as the time interval from the date of administration of first ICB containing treatment until progression or death. Alive patients without disease progression were censured at the date of data cut off. The log-rank test was used to assess differences in survival between groups. Multivariate Cox regression analysis was conducted to compensate for potential confounders including sex, age, smoking status, PS, stage at inclusion, histology, line of treatment, PD-L1 and TMB. For the merged PFS cohort, multivariable Cox regression analysis was also conducted to compensate for the potential confounding factors sex, age and smoking status. Statistical significance was set at *p* < 0.05, and no adjustments were made for multiple comparisons. Data analysis was conducted using IBM SPSS Statistics version 27 and GraphPad Prism version 9.

## Results

### Classification model for identification of variants

**Fig. 2:**
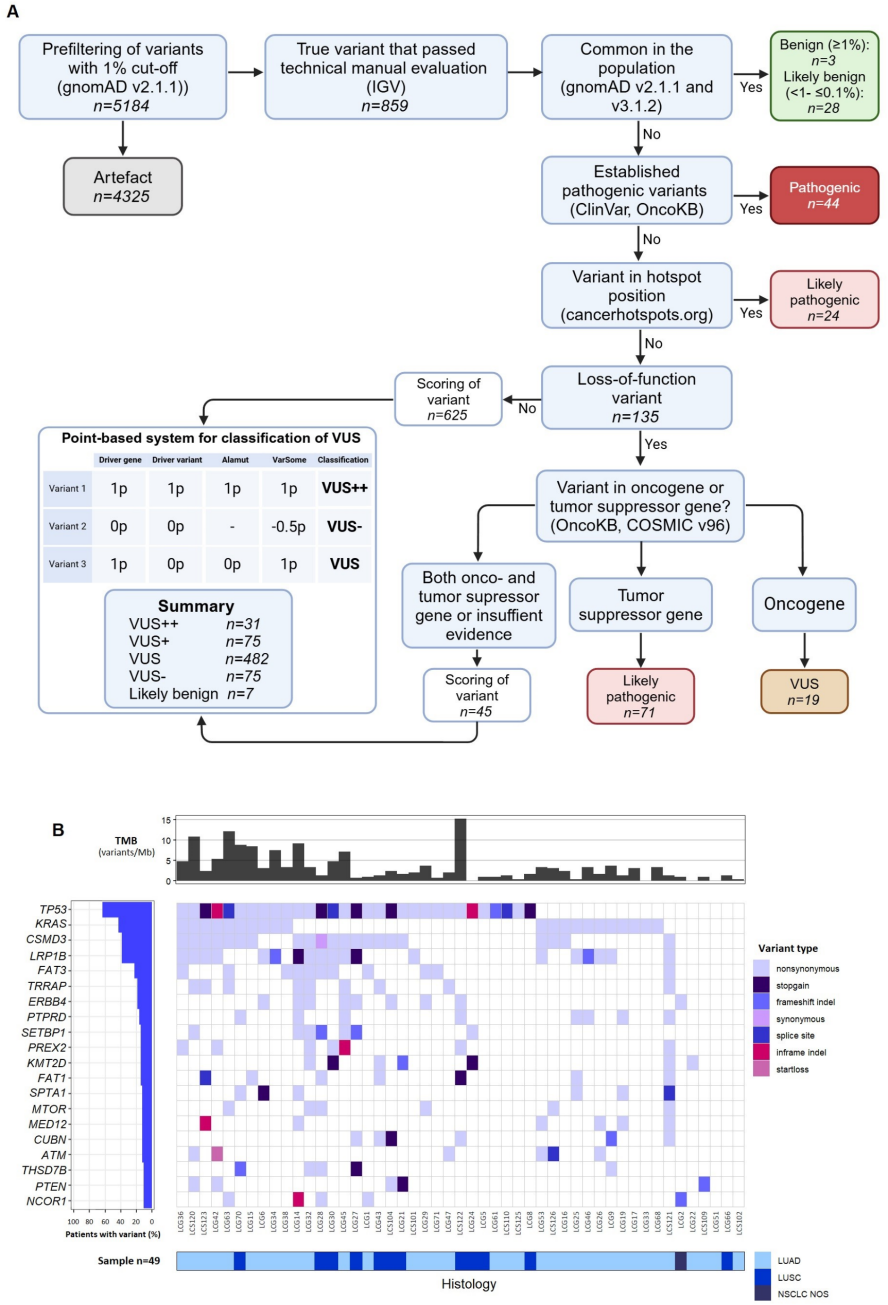
Overview of method workflow and results. (**A**) Classification and interpretation model. (**B**) Waterfall plot showing the most frequently mutated genes in the patient cohort in categories pathogenic (P), likely pathogenic (LP), and variant of unknown significance (VUS; VUS + + and VUS+). The top plot shows the tumor mutational burden (TMB) for each sample and the bottom plot shows the histological subtype. The waterfall plot was constructed in R v4.3. Abbreviations used: LUAD = lung adenocarcinoma and LUSC = lung squamous cell carcinoma. TMB = tumor mutational burden

We established a classification model for the integration of theoretical prediction tools and database information to interpret variants from a large NGS screening panel into different classification categories in genes commonly mutated in NSCLC (Fig. 2A). We further evaluated the predictive biomarker potential of these gene variants as single variants or in combinations as co-variants. In addition, mutational signature analysis was performed on the NGS data for all the patients.

The result of the molecular analysis including the most commonly mutated genes, type of mutations and histology are presented in Fig. 2B. Among 49 patients analyzed we identified and classified 141 DNA variants as P or LP (Additional file 5). The VUS categories included 607 variants (VUS++, VUS + and VUS) in total and ranged from 0 to 71 variants per sample. A total of total of 106 variants in the categories VUS + + and VUS+(31 VUS++, 75 VUS+) were identified. In addition, 501 variants were classified as VUSs (Additional file 3 and 6).

The most frequently identified pathogenic variant was *KRAS* (34%). Twenty-nine patients (59%) had known pathogenic variants in either *KRAS* (p.(G12C), p.(G12A), p.(G12V), p.(G13C), p.(Q61L), and p.(Q61H)), *IDH1 (*p.(R132C)), *MET* (exon 14 skipping), *ROS1* (p.(G2028R/G2032R)), *PIK3CA (*p.(E545K)), *EGFR* (p.(G719C)) or *KIF5B-RET* gene fusions identified through the initial clinical NGS panel (Additional file 1). With the established workflow we identified P and LP variants in several additional genes including *TP53* (32 variants), *LRP1B* (6 variants), *ARID1A* (6 variants), *ATM* (4 variants), *KEAP1*,* BRCA1* and *BRCA2*. Among VUS (VUS++, VUS + and VUS) 28 variants were identified in *CSMD3*, 16 variants in *LRP1B* and 13 variants in *FAT3*. VUS ++, VUS + also included variants in *KEAP1*, *DNMT3A*, *TSC2*, *LZTR1* and *POT1*. One of the most common genes identified with LP and strong VUSs was *LRP1B* (Additional file 6 and 7). One non-responding patient with an *LRP1B* variant also had two variants in *KEAP1* (classified as VUS+) and another non responder who carried an *LRP1B* variant had an additional *STK11* variant. One responder had two *LRP1B* variants and a *KEAP1* variant that was classified as VUS. The *KEAP1* variant, was however localized in the last nucleotide of the last exon in the main transcript (NM_203500.2:c.1875 A > C, p.(*625Cysext*48)) and therefore the variant might not affect the protein.

The genes with the most commonly combined alterations were *CSMD3* and *TP53*, which were found in 17 patients (35%). Combination of DNA variants in *KRAS* and *LRP1B* were found in 11 patients (22%) (Additional file 8). In addition, combinations of *TP53* and *KRAS* variants were found in 10 patients (20%). Combinations of *TP53* and *LRP1B* were also common (27%, 13 patients), and the combination of *KRAS*, *LRP1B* and *TP53* was found in 6 patients (12%).

### Patient demographics and clinical characteristics

The median age of the patients were 72 years, 89% were ever smokers (former or current) and the majority the patients were female (55%). At inclusion, most patients had an ECOG performance status of 1 (61%), and adenocarcinoma was the most common histological subtype (67%). The majority of patients had stage IV disease at inclusion (91%) and 71% of all the patients received ICB in a first-line treatment setting. More than half of the patient cohort received combined treatment with chemotherapy and ICB (59%), about a third received ICB monotherapy (32%) and a smaller proportion received adjuvant ICB (8%). The median follow-up time was 31 months (Additional file 1). Patient LCS125 experienced a change in treatment before progression and was therefore excluded from PFS analysis.

### Single variants in ***LRP1B*** and ***KRAS*** are predictive biomarkers of survival outcomes following ICB treatment

**Fig. 3:**
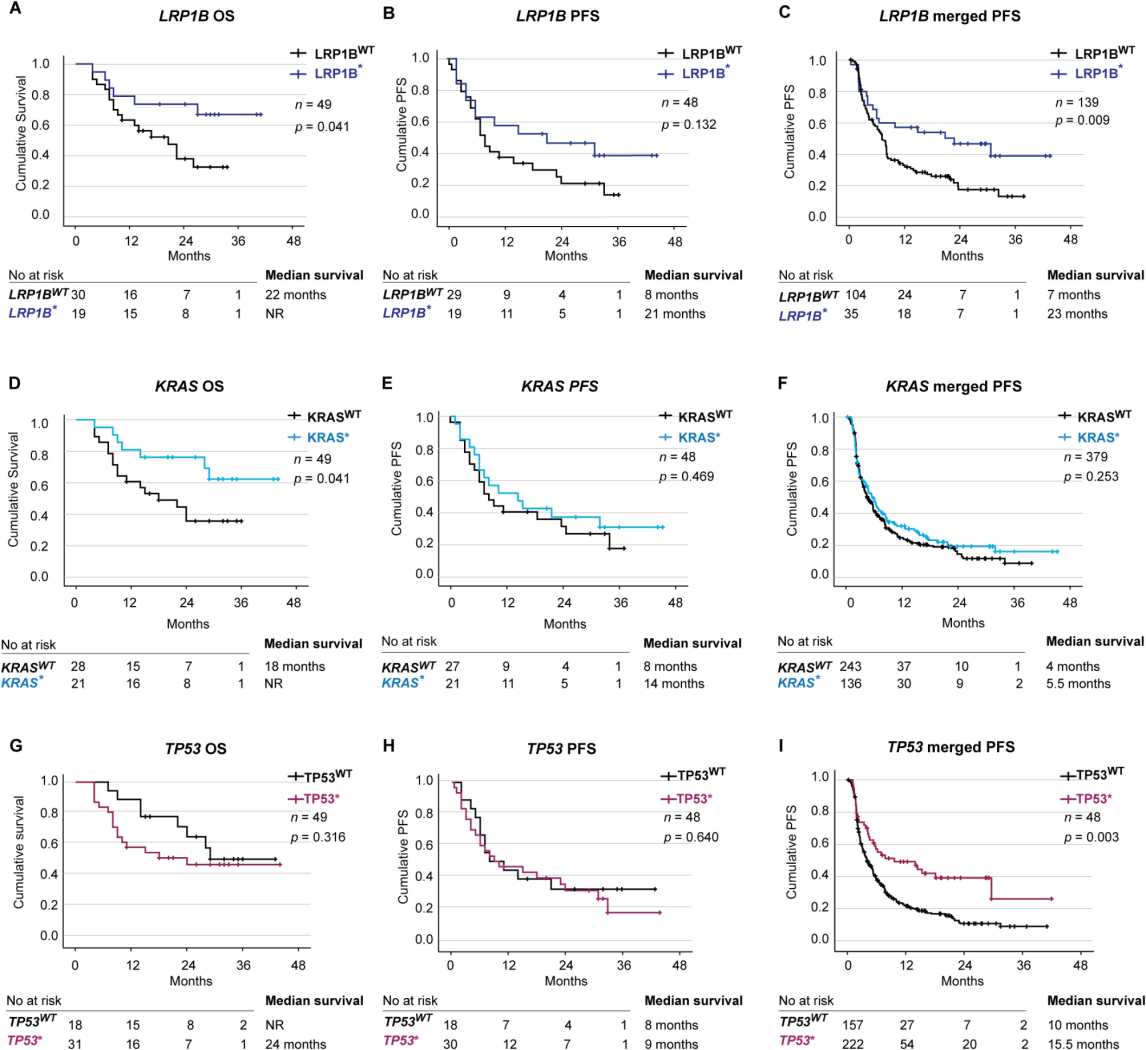
Single variants as predictive biomarkers. Kaplan-Meier estimates comparing overall survival (OS) (**A**) and progression free survival (PFS) (**B**) and merged progression free survival (**C**) stratified on *LRP1B* status. Kaplan-Meier estimates comparing overall survival (**D**) and progression free survival (**E**) and merged progression free survival (**F**) stratified on *KRAS* status. Kaplan-Meier estimates comparing overall survival (**G**) and progression free survival (**H**) and merged progression free survival (**I**) stratified on *TP53* status. *DNA variant classified as P, LP, VUS++, VUS + and VUS

Based on the most common identified molecular variants in the cohort, we analyzed *LRP1B*, *KRAS*,* TP53* and *CSMD3* as single variant predictive biomarkers for OS and PFS. The presence of an *LRP1B* DNA variant (LP, VUS++, VUS + or VUS) was beneficial for OS, with a median OS not reached vs. 22 months (*p* = 0.041), no *LRP1B* variants were classified as VUS- likely benign or benign (Fig. 3A and Additional file 7). Multivariate Cox regression analysis revealed that *LRP1B* was an independent predictor of improved OS (HR 0.280, 95% CI 0.096–0.819; *p* = 0.020) (Additional file 9 A). Median PFS was better for patients with *LRP1B* variants with 21 months vs. 8 months, but the difference did not reach statistical significance (*p* = 0.132) (Fig. 3B). In the merged PFS group, patients with *LRP1B* variants had a significantly better median PFS 23 months vs. 7 months (*p* = 0.009) (Fig. 3C). In multivariate Cox regression analysis, *LRP1B* was an independent predictor of prolonged merged PFS (HR 0.515, 95% CI 0.299–0.885; *p* = 0.016) (Additional file 9 B).

Having *KRAS* pathogenic variants significantly improved survival with a median OS not reached vs. 18 months (*p* = 0.041) (Fig. 3D). Multivariate Cox regression analysis revealed that solely *KRAS* pathogenic variants were not an independent factor for better OS (HR 0.422, 95% CI 0.143–1.247; *p* = 0.119) (Additional file 9 C). Numerically the median PFS was better for patients with *KRAS* pathogenic variants 14 months vs. 8 months, but the difference was not statistically significant (*p* = 0.469) (Fig. 3E). In the merged PFS cohort, there was no significant difference between patients with *KRAS* pathogenic variants and wild-type *KRAS* (*p* = 0.253) (Fig. 3F).

With regard to *TP53*, there was no impact on OS or PFS in our cohort alone (Fig. 3G and H). However, in the merged PFS group, patients harboring *TP53* pathogenic variants had significantly longer PFS, with a median of 15.5 vs. 10 months (*p* = 0.003) (Fig. 3I). Multivariate Cox regression analysis revealed that *TP53* was an independent predictor of prolonged merged PFS (HR 0.724, 95% CI 0.552–0.950; *p* = 0.020) (Additional file 9 D).

Single *CMSD3* variants did not impact OS or PFS in the cohort and the variants found were mainly benign (Additional file 10 A and 10B).

### Combined variants ***LRP1B*** and ***KRAS*** a better predictive biomarker

Next, we investigated the impact of the combination of above variants on OS and PFS.

**Fig. 4:**
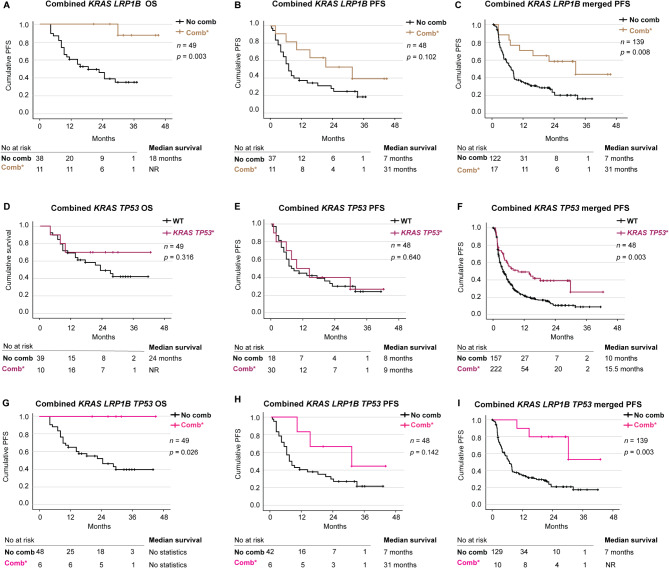
Co-variants as predictive biomarkers. Kaplan-Meier estimates comparing overall survival (OS) (**A**), progression free survival (PFS) (**B**) and merged progression free survival (**C**) stratified on co-variant *KRAS LRP1B* status. Kaplan-Meier estimates comparing overall survival (**D**), progression free survival (**E**) and merged progression free survival (**F**) stratified on co-variant *KRAS* and *TP53* status. Kaplan-Meier estimates comparing overall survival (**G**), progression free survival (**H**) and merged progression free survival (**I**) stratified on co-variant *KRAS*, *LRP1B* and *TP53* status. *DNA variant classified as P, LP, VUS++, VUS + and VUS

We found that having combined *KRAS* and *LRP1B* variants significantly improved OS, with a median not reached vs. 18 months (*p* = 0.003) (Fig. 4A). Multivariate Cox regression analysis revealed that the combination of *KRAS* and *LRP1B* was an independent predictor of improved OS (HR 0.062, 95% CI 0.008–0.493; *p* = 0.009) (Additional file 9 E). When analyzing PFS, the median time to progression was 31 months for combined *KRAS* and *LRP1B* variants vs. 7 months (*p* = 0.102) (Fig. 4B). In the merged PFS group, the combination of *KRAS* and *LRP1B* variants had a median PFS of 31 vs. 7 months, and the difference was statistically significant (*p* = 0.008) (Fig. 4C). Multivariate Cox regression revealed that the combination of *KRAS* and *LRP1B* was an independent predictive factor for improved merged PFS (HR 0.412, 95% CI 0.188–0.902; *p* = 0.027) (Additional file 9 F).

Furthermore, we stratified patients into four groups: wild-type *KRAS* and wild-type *LRP1B*, *KRAS* variant with wild-type *LRP1B*, *LRP1B* variant with wild-type *KRAS*, or the combination of *KRAS* and *LRP1B* variants. The stratification was done to investigate how patients with wild-type of *KRAS* or *LRP1B* or variants in these genes responded compared to patients having wild-type of both or combination of both. The results demonstrated that the combination of *KRAS* and *LRP1B* variants seemed to be driving response, as the group *LRP1B* variant with wild-type *KRAS* had shorter OS and PFS compared to all the other groups. *KRAS* variant with wild-type *LRP1B* displayed slightly longer OS and PFS, but still shorter than wild-type of both and combination of both. The results demonstrated that the combination of *KRAS* and *LRP1B* variants were associated with improved OS, with a median not reached in comparison with patients with no variants 22 months (median). This enhancing the results described earlier that patients with the combination of *LRP1B* and *KRAS* variants respond significantly better to ICB. For those with only *KRAS* variants the OS was 14 months and for those with only *LRP1B* variants the OS was 9 months (*p* = 0.028) (Additional file 11 A). When analyzing PFS, the same trend was observed, but was not statistically significant for the cohort (*p* = 0.379) (Additional file 11 B). In the merged group, the combination of *KRAS* and *LRP1B* variants was beneficial for PFS, with a median of 31 months, whereas the group without the variants had a PFS of 7 months, only *KRAS* variants had a PFS of 8 months, and only *LRP1B* variants had a PFS of 5 months (*p* = 0.038) (Additional file 11 C).

Combined *KRAS* and *TP53* pathogenic variants did not significantly impact OS, with a median not reached vs. 24 months (*p* = 0.316) (Fig. 4D). Neither regarding PFS did we observe any difference between patients with combined *KRAS* and *TP53* pathogenic variants with a median PFS of 9 vs. 8 months (*p* = 0.640) (Fig. 4E). However, when analyzing the merged PFS group, there was a significant improvement in survival with the combined *KRAS* and *TP53* pathogenic variants with a median of 15.5 months vs. 10 months (*p* = 0.003) (Fig. 4F).

Further we investigated the impact of triple combination of *KRAS*,* LRP1B* and *TP53* on survival. We found that all six patients harboring the triple combination were alive at last follow-up and OS was significantly higher than that of patients without the triple combination (*p* = 0.026) (Fig. 4G), no median OS could be calculated. The PFS was 31 months for the triple combination group vs. 7 months although the difference was not significant (*p* = 0.142) (Fig. 4H). In the merged PFS, median was not reached for the triple combination group vs. 7 months, and the difference was significant (*p* = 0.003) (Fig. 4I). Multivariate Cox regression revealed that harboring the triple combination was an independent predictor of improved merged PFS (HR 0.221, 95% CI 0.067–0.728; *p* = 0.013) (Additional file 9 G).

To investigate the impact of *TP53* in the triple combination, we stratified patients in the *KRAS-LRP1B* combination cohort (*n* = 17) in the merged PFS cohort according to *TP53* status. Our analysis revealed that the presence of a *TP53* variant was clearly beneficial, with median not reached vs. 6.5 months to progression, respectively, and the difference was significant (*p* = 0.047) (Additional file 11D).

There were no other established biomarkers that were enriched in patients with *LRP1B* variants classified as P, LP, VUS + + or VUS+. Interestingly, in two patients we found that co-variants of *LRP1B* and *KRAS* together with *KEAP1* or *STK11* did not negatively impact the response. (Additional file 7).

### PD-L1 expression alone and in combination as a predictive biomarker

**Fig. 5:**
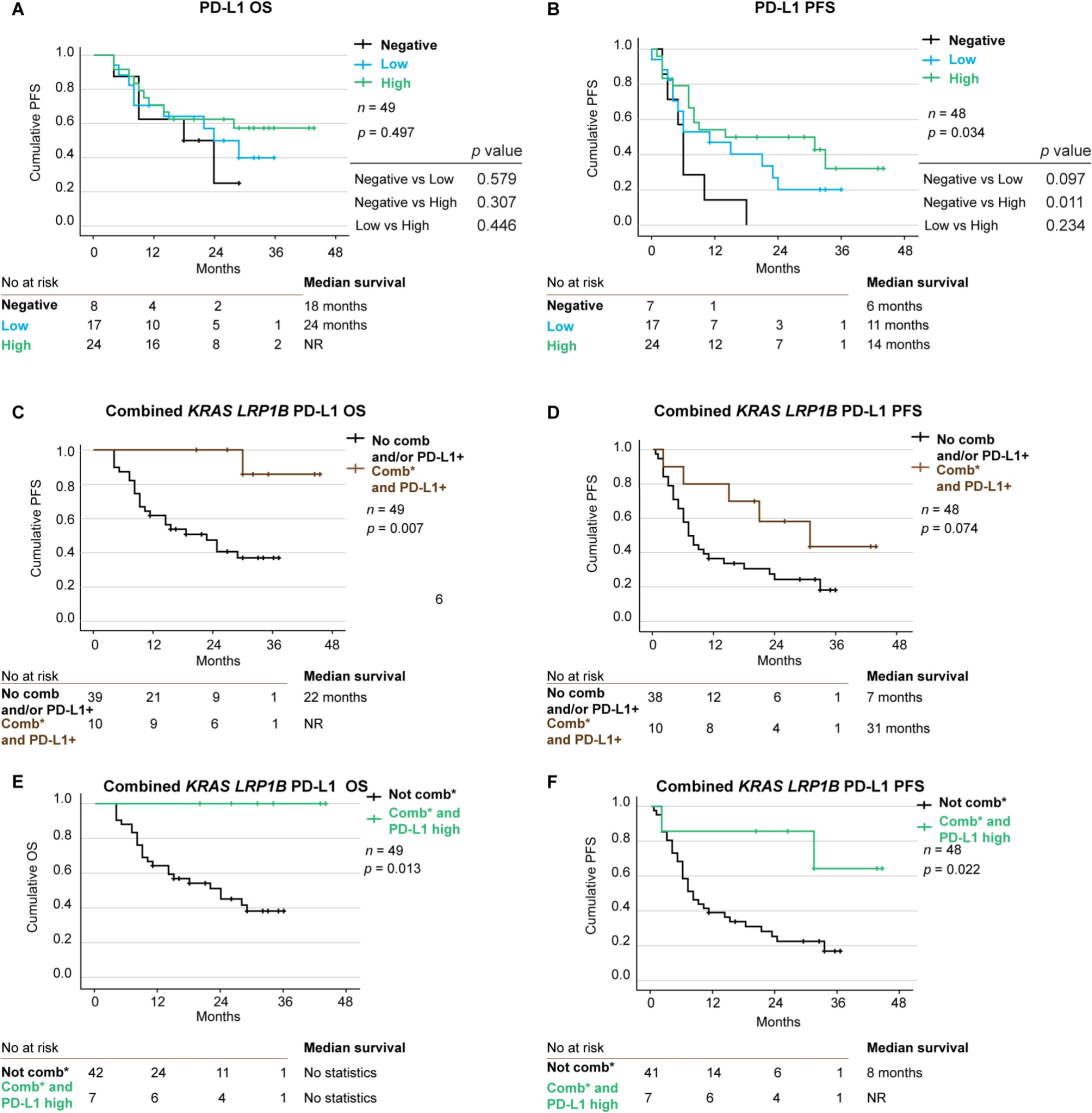
PD-L1 status and co-variants as predictive biomarkers. Kaplan-Meier estimates comparing overall survival (OS) (**A**) and progression free survival (PFS) (**B**) stratified on PD-L1 status. Kaplan-Meier estimates comparing overall survival (**C**) and progression free survival (**D**) stratified on combined *KRAS LRP1B* and PD-L1 positive status. Kaplan-Meier estimates comparing overall survival (**E**) and progression free survival (**F**) stratified on combined *KRAS*,* LRP1B* and PD-L1 high status. *DNA variant classified as P, LP, VUS++, VUS + and VUS

PD-L1 grade (negative, low or high) is the current standard biomarker approved for ICB treatment in Europe. Hence, we also analyzed the impact of PD-L1 grade on OS and PFS alone and combined with *KRAS* and *LRP1B* variants. PD-L1 score had no impact on OS but a significant impact on PFS, with median PFS 6 months for the negative group, 11 months for the low group and 14 months for the high group (*p* = 0.034) (Fig. 5A and B).

Notably, having PD-L1-positive (> 1%) tumors combined with variants in *KRAS* and *LRP1B* was predictive of better survival, with a median not reached vs. 22 months, (*p* = 0.007) (Fig. 5C). Multivariate Cox regression revealed that PD-L1-positive tumors combined with variants in *KRAS* and *LRP1B* was an independent predictor of improved OS (HR 0.087, 95% CI 0.011–0.688; *p* = 0.021) (Additional file 9 H). For PFS, there was a clear difference in the median time to progression 31 months vs. 7 months, but the difference was not statistically significant (*p* = 0.074) (Fig. 5D). However, multivariate Cox regression revealed that PD-L1-positive tumors combined with *KRAS* and *LRP1B* DNA variants was an independent predictive factor for improved PFS (HR 0.334, 95% CI 0.113–0.984; *p* = 0.047) (Additional file 9 I).

**Fig. 6:**
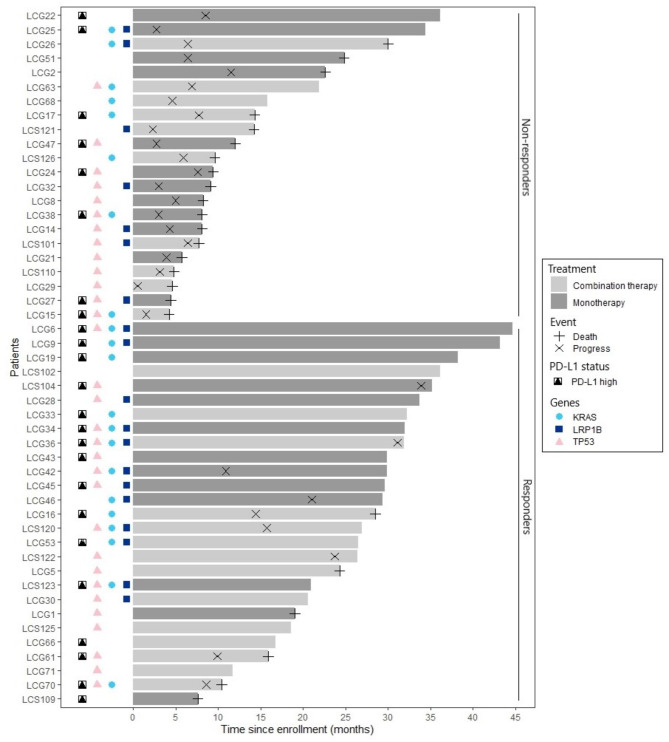
Swimmer’s plot displaying patients with specific characteristics. Swimmer’s plot where patients are divided into non-responders and responders based on response to immune checkpoint blockade. Color of bars show treatment regime and length of bars present the survival time in months from inclusion in the study. In case of progressive disease or death, this is marked with crosses. PD-L1 high (≥ 50%) is denoted. Patients with genetic alterations in *KRAS*,* LRP1B* and *TP53* are marked, respectively

In the next step we analyzed the impact of high PD-L1(≥ 50%) expression in combination with variants in *KRAS* and *LRP1B*. We found that having high PD-L1 expression and combined variants in *KRAS* and *LRP1B* was clearly beneficial for OS with all patients being alive and median not reached (*p* = 0.013) (Fig. 5E). Similarly, PFS median not reached in the PD-L1-high score group vs. 8 months (*p* = 0.022) (Fig. 5F). The combination of *KRAS*, *LRP1B* and PD-L1 high is clearly enriched in the responding group of the cohort and there was only one single patient harboring all three in the non-responding group (Fig. 6). Notably the triple combination with *KRAS*,* LRP1B* and *TP53* was not present at all in the non-responder group (Fig. 6).

## Discussion

Pathogenic oncogenic driver variants are the current standard when selecting targeted treatment options for NSCLC patients. Loss of function variants in tumor suppressor genes is not routinely assessed in the clinic, despite accumulating evidence for the importance of co-variants identified in tumor analysis, both for prognosis and treatment response in NSCLC [33–37, 59]. This is mainly due to the lack of studies showing the clinical impact of co-variants. Additionally, with the ongoing implementation of a comprehensive variant analysis in clinical routine, there is a critical need to understand how to utilize the additional information provided by the identification of a large number of DNA variants using genomics screening methods where a large number of genes are included.

In this study, we present a combination of bioinformatic tools that can be used to assess pathogenicity of identified variants from a genomic screening analysis, by integrating clinical available data with the evidence on a molecular level in a novel developed model. Applying this model, we show that co-variants of these three genes (*KRAS*, *LRP1B* and *TP53*) can clearly predict clinical response and survival outcomes.

Although current bioinformatic pipelines manage to call various types of variants from all forms of NGS data, a significant bottleneck remains in the interpretation. One of the primary challenges is the accurate interpretation of the missense variants that are most likely to disrupt protein function. Despite the availability of various disease- and treatment-associated databases like OncoKB, ClinVar, large normal population-based databases like gnomAD and other prediction tools, identifying pathogenic driver variants remains challenging, particularly for genes with unknown functional mechanisms.

We weighed the available evidence through a point-based evaluation system to differentiate the VUSs, potentially indicating their pathogenicity. This increased the understanding of the impact of variants in less well-known genes and better identified DNA variants affecting the treatment response in combination with known pathogenic drivers such as *KRAS*. We identified 78% P/LP variants in oncogenes and tumor suppressor genes and identified VUSs (VUS++, VUS+) with pathogenic potential. Co-variants in the same gene may be associated with different outcomes in their predictive or prognostic effect depending on the localization of the variant in the gene and the type of variant (truncating, splice variant, missense etc.). The detailed classification of VUSs in this study aimed to better identify potentially modifier variants and the impact of co-variants on the response. Hence, strong molecular VUSs, based on their interpretation and classification, were included throughout the analysis.

In our analysis *KRAS*,* LRP1B* and *TP53* were three of the most common genes with variants identified. *KRAS* is a well-known driver, and *TP53* is the most common inactivated tumor suppressor gene in NSCLC, whereas *LRP1B* is a lesser-known tumor suppressor gene. Having a combination of all three variants is in our cohort was predictive of better response to ICB-containing treatment. The addition of PD-L1 positivity and especially high PD-L1 seems to be even more beneficial.

Alfaro-Murillo and Townsend [60] developed a computational model for the evaluation of somatic variants that included several parameters affecting which possible routes of sequential genetic alteration occurrence could be used in oncogenesis. The four most common driver genes in lung adenocarcinoma patients within NSCLC, *TP53*,* LRP1B*,* KRAS*, and *STK11*, were included in the analysis. The model could predict the interaction, competition, and selection benefits of different DNA variants in these genes. The results from the analysis revealed that the combination of variants in *TP53* and *LRP1B* strongly selected for high-effect driver variants in *KRAS*. These results on the effect of the co-variants on the *LRP1B*,* TP53* and *KRAS* genes correlated with our results and impacted the response to ICB.

When exploring single variants as predictive biomarkers we found *LRP1B* alone to improve OS and PFS in our cohort. *LRP1B* has been found to be associated with improved outcomes in multiple cancer types, including NSCLC, with ICB treatment [38, 39, 61–63]. Brown et al. and Wang et al. identified *LRP1B* variants as potential biomarkers among ICB treated patients in accordance with our study. However, in these studies only a limited use of bioinformatic tools and databases were included and a more detailed interpretation and classification was not performed especially for the large group of VUS variants.

The *LRP1B* gene encodes a member of the low-density lipoprotein receptor family and is located on chromosome 2. Human LRP1B is highly expressed in normal tissues, including the lung. The large gene size (approximately 1.90 Mbps) has been challenging for performing functional studies. Cell proliferation, migration, apoptosis and endocytosis are some processes that have been linked to LRP1B and to proteins that interact with LRP1B [64–67]. LRP1B has been found to have a growth suppressive function, which is correlated with its suggested role as a tumor suppressor gene [68–71].

Analysis of available NSCLC datasets in cBioPortal revealed that *LRP1B* was only included in one gene panel or in studies where whole-genome or whole exome sequencing was used (Additional file 12). In these studies, variants in *LRP1B* were detected in 32.7% of patients (849 of 2597 patients). *KRAS* was detected in 25.7% of patients (668 of 2597 patients). The combination of *LRP1B* and *KRAS* was detected in 13.3% of the patient population (346 of 2597 patients), and the triple combination of *KRAS*, *LRP1B* and *TP53* was detected in 5.5% of the patients (142 of 2597). These numbers were slightly higher in our cohort, where 22.4% (11 of 49 patients) had a genetic variant in both *LRP1B* and *KRAS*, and the combination of *KRAS*, *LRP1B* and *TP53* was found in 12.2% (6 of 49 patients). The difference may be explained by the fact that our study only included patients eligible for ICB treatments and thus excluding patients with targetable alterations. Our findings suggest that identifying such genetic profiles may help identify patient subgroups likely to benefit from precision medicine approaches.

Comparing the outcomes for patients with *LRP1B* LP/P variants who received ICB to those with VUS (according to ClinVar), both OS and PFS were significantly better [38]. These results support the value and importance of variant interpretation for understanding the impact of new variants foremost as predictive biomarkers of response to immunotherapy. *LRP1B* mutation has been associated with increased immune cell infiltration and elevated expression of immune-related genes in NSCLC patients with adenocarcinoma treated with ICB, suggesting a possible role in the improved response to ICB [72, 73]. Furthermore, knockdown of *LRP1B* has been shown to induce inflammation through the IL-6-JAK-STAT3 pathway [69]. However, its function both in normal tissue and in cancer is still poorly understood and additional functional studies are needed to fully understand the connections between *LRP1B*, the immune system and the response to ICB. Importantly, while previous retrospective studies have implicated LRP1B primarily as a prognostic biomarker [38, 63, 74], our prospective study suggests LRP1B variants as predictive biomarkers for ICB response. Furthermore, prior studies have not examined the interaction between LRP1B and KRAS mutations, a key focus of our study.

Variants in *KRAS* as a sole variant was not significantly associated with improved survival in the multivariate analysis, as previously shown [27]. This might reflect both the heterogeneity among patients with different *KRAS*-variants as well as the importance of co-variants. Here, we show that this effect of improved outcomes after ICB is primarily driven by the combination of mutations in *LRP1B* with *KRAS*, which has not been described before. *KRAS* variants are strongly linked to smoking and high TMB [75], and *LRP1B* variants might also be associated with high TMB [39, 74]. In Wang et al. no correlation between co-variants in *LRP1B* ad *KRAS* and response was found, however in their large retrospective study only 2% of the patients had a combination of *LRP1B* and *KRAS*, compared with our study where 22.4% of the patient had this combination, highlighting the importance of evaluating combination of variants in different populations.

Patients with non-synonymous variants in *TP53* have shown longer PFS with ICB monotherapy compared with patients that are wild type for *TP53* when analyzing cBioPortal data and the same tendency was seen in a combination therapy cohort. Patients with co-variants in *KRAS* and *TP53* also had a positive correlation with response and variants in *KEAP1* and *STK11* together with variants in *KRAS* and *TP53* trends towards better prognosis compared with tumors that were wild-type for *KRAS* and *TP53*. In a recent study *KRAS* (G12C)/*TP53* co-variants correlated with long-term response to monotherapy treatment suggesting that the type of *KRAS* variant might be important [76, 77]. Different variant types in several genes have also been found to correlate differently to treatment; for *TP53*, R175H had a negative impact on the response to immunotherapy in several metastatic solid cancers [78]. Truncating variants in *KEAP1* including exon1 and 2 have been associated with worse outcome than variants in other parts of the coding region [79].

This study, to our knowledge, is the first to report that harboring a combination of variants in the *KRAS*,* LRP1B* and *TP53* can clearly be beneficial for the response to ICB and could be considered a potential predictive biomarker for NSCLC patients. Why the combination of variants in the *KRAS*,* LRP1B* and *TP53* genes are beneficial for ICB treatment needs further functional investigation. Limitations of this study include the relatively small size of the cohort, and the results should be further validated in larger cohorts. The study was also limited to panel analysis and did not include analysis of the whole exome or genome, which could also explain a difference in the correlation between a high TMB and variants in *LRP1B* found in previous studies [74]. Functional analyses are also necessary to elucidate the interplay between *KRAS*, *LRP1B*, and *TP53* to guide treatment decisions.

## Conclusions

In our well-characterized prospective cohort of patients, we assessed genetic biomarkers to predict treatment response to ICB. Our findings offer a potential explanation for the previously inconsistent reports regarding the response of patients with *KRAS* variants to ICB. Specifically, the possibility that combinations of *KRAS* and other genetic variants may significantly influence treatment response emphasizes the importance of comprehensive genetic testing and analysis. Notably in this study, the combination of *KRAS* and *LRP1B* variants emerges as a clear promising prognostic biomarker, with the potential addition of *TP53* to enhance prognostic accuracy. However, larger cohort studies and functional analyses are necessary to elucidate the interplay between *KRAS*, *LRP1B*, and *TP53* to guide treatment decisions. This study highlights the critical importance of including *LRP1B* in targeted gene panels for clinical routine testing to improve patient stratification and therapeutic outcomes.

## Electronic supplementary material

Below is the link to the electronic supplementary material.

**Supplementary Material 1**: **Additional file 1.xlsx– Patient characteristics**: Table showing the baseline characteristics of the included patients presented in number (n) and percent (%).

**Supplementary Material 2**: **Additional file 2.xlsx– List of genes screened for in panel**: Displays a list of genes that were tested within the larger next-generation sequencing panel. Oncopanel All-In-One v2.8.

**Supplementary Material 3**: **Additional file 3.pdf - An evaluation system used to classify variants**: Specifically, to classify Loss-of-Function (LoF) variants in genes with insufficient or conflicting evidence about tumor-suppressing or oncogenic characteristics and also non-LoF variants. The system consists of eight parameters each giving a certain amount of points. More points suggest more pathogenic influences. Parameters with green background are not checked for all variants, only under certain circumstances. The amount of points a variant receives represents a classification where ≤-1 *p* = likely benign, -0.5 *p* = VUS-, 0–2 *p* = VUS, 2.5-3 p = VUS + and ≥ 3.5 *p* = VUS++.

**Supplementary Material 4**: **Additional file 4.pdf– Mutational signatures for LCG48**: Profile of mutational signature analysis for patient LCG48. LCG48 was an outlier and presented a profile consisting of 92% SBS7a and SBS7b (cosine similarity of 0.984). Signatures associated with UV light exposure. Abbreviation used: SBS = Single base substitution.

**Supplementary Material 5**: **Additional file 5.xlsx - Summary of variants classified as pathogenic and likely pathogenic**: Shows a list of patients and each variant classified as pathogenic or likely pathogenic. The table also includes a column of which variants were found in the broader next-generation sequencing panel.

**Supplementary Material 6**: **Additional file 6.xlsx– Summary of variants classified as VUS**,** VUS + and VUS++**: Shows a list of patients and each variant classified as VUS, VUS + or VUS++. The table also includes the results from the point-based evaluation system. Abbreviations used: VUS = variant of unknown significance.

**Supplementary Material 7**: **Additional file 7.xlsx– Summary of variants for patients with an*LRP1B*-variant**: Presents a list of patients with an LRP1B-variant together with other variants classified as pathogenic, likely pathogenic, VUS + + or VUS+. One column presents the patient’s clinical response to treatment. Abbreviations used: VUS = variant of unknown significance.

**Supplementary Material 8**: **Additional file 8.xlsx– Co-variants among the most commonly mutated genes within the cohort**: Combination of genes co-mutated within ten or more patients have dark blue backgrounds.

**Supplementary Material 9**: **Additional file 9 A-I-.xlsx- Multivariate cox-regression analyses**.

**Supplementary Material 10**: **Additional file 10.pdf–*CSMD3***: Kaplan-Meier estimates comparing overall survival (A) and progression free survival (B) stratified on *CSMD3* status.

**Supplementary Material 11**: **Additional file 11.pdf– Single and combined variants as predictive biomarkers**: Kaplan-Meier estimates comparing overall survival (A) and progression free survival (B) and merged progression free survival (C) stratified on having neither, one of or combined KRAS* and LRP1B* variants. Kaplan-Meier estimates comparing merged progression free survival (D) for the combined KRAS* and LRP1B* variants population stratified on TP53* status. *DNA variant classified as P, LP, VUS++, VUS + and VUS.

**Supplementary Material 12**: **Additional file 12.xlsx– List of publications with*LRP1B***: The NSCLC studies in cBioPortal where *LRP1B* were tested.

## Data Availability

Availability of data and materials The datasets supporting the conclusions of this article are included within the article and its additional files.

## Abbreviations

ICB: Immune checkpoint blockade
Indels: Insertions and deletions
LP: Likely pathogenic
Mb: Megabase
NGS: Next-generation sequencing
NSCLC: Non-small cell lung cancer
OS: Overall survival
P: Pathogenic
PD-L1: programmed death-ligand 1
PFS: Progression-free survival
SNVs: Single nucleotide variants
TMB: Tumor mutational burden
TPS: Tumor proportional score
VUS: Variant of uncertain significance
