# Supplementary material for: Comprehensive genetic variant analysis reveals combination of KRAS and LRP1B as a predictive biomarker of response to immunotherapy in patients with non-small cell lung cancer
Source: J Exp Clin Cancer Res. 2025 Feb 27;44:75. doi: 10.1186/s13046-025-03342-6 (PMC11866712; doi:10.1186/s13046-025-03342-6)
Supplement: Supplementary file 3 — Supplementary Material 3: Additional file 3.pdf - An evaluation system used to classify variants: Specifically, to classify Loss-of-Function (LoF) variants in genes with insufficient or conflicting evidence about tumor-suppressing or oncogenic characteristics and also non-LoF variants. The system consists of eight parameters each giving a certain amount of points. More points suggest more pathogenic influences. Parameters with green background are not checked for all variants, only under certain circumstances. The amount of points a variant receives represents a classification where ≤-1 p = likely benign, -0.5 p = VUS-, 0–2 p = VUS, 2.5-3 p = VUS + and ≥ 3.5 p = VUS++. [file 13046_2025_3342_MOESM3_ESM.pdf]

Additional file 3. An evaluation system used to classify variants.

		Score (points)						
**#**	**Parameter**	**+3**	**+1.5**	**+1**	**+0.5**	**0**	**-0.5**	**-1**
**1**	**Number of entries** (COSMIC[1])	≥50	50>x>10			≤10		
**2**	**Driver gene** (CancerGeneCensus[2])			Yes		No		
**3**	**Predicted driver gene** (Cancer-genes.org[3], IntOGen[4])*			Yes		No		
**4**	**Interpreted variant effect** (CancerGenomeInterpreter[5])			Yes		Passenger, blank or error		
**5**	**Described in functional studies** (VarSome[6] (including CIViC[7], UniProt[8], DoCM[9] and Mastermind[10]), LitVar[2] [11], dbSNP[12])			Pathogenic, disease causing or drug resistant	Likely pathogenic	No data available		Benign
**6**	**ACMG classification** (VarSome (including REVEL, MetaRNN, BayesDel addAF, Polyphen))			Pathogenic or likely pathogenic with VarSome-score of 9p	Likely pathogenic or VUS+	VUS	Likely benign	Benign
**7**	**Bayesdel addAF algorithm** (VarSome)**				Strong to moderate pathogenic			
**8**	**Conservation of amino acid and Grantham distance** *** (AlamutVisualPlus v1.4 (also including AlignGVGD, PolyPhen2, SIFT (v6.2.0), MutationTaster (2021))			Highly conserved amino acid and moderate to high Grantham distance	Predicted to affect splicing >10 %			

*If not present in parameter 2

**Only checked if parameter 6 described the variant as likely benign based on the gene not having any pathogenic variants.

***Checked if total score above 2 from other parameters

Specifically to classify Loss-of-Function (LoF) variants in genes with insufficient or conflicting evidence about tumor-suppressing or oncogenic characteristics and also non-LoF variants. The system consists of eight parameters each giving a certain amount of points. More points suggest more pathogenic influences. Parameters with green background are not checked for all variants, only under certain circumstances. The amount of points a variant receives represents a classification where ≤-1 p = likely benign, -0.5 p = VUS-, 0-2 p = VUS, 2.5-3 p = VUS+ and ≥ 3.5 p = VUS++.

[1] COSMIC v.96, https://cancer.sanger.ac.uk/cosmic
[2] CancerGeneCensus (COSMIC v.96), https://cancer.sanger.ac.uk/census
[3] Cancer-genes.org, cancer-genes.org
[4] IntOGen, https://www.intogen.org/search
[5] CancerGenomeInterpreter (CGI), https://www.cancergenomeinterpreter.org/home
[6] VarSome, https://varsome.com/
[7] Clinical Interpretation of Variants in Cancer (CIViC), https://civicdb.org/welcome
[8] UniProt, https://www.uniprot.org/
[9] Database of Curated Mutations (DoCM), http://docm.info/
[10] Mastermind, https://mastermind.genomenon.com/
[11] LitVar[2], https://www.ncbi.nlm.nih.gov/research/litvar2/
[12] dbSNP, https://www.ncbi.nlm.nih.gov/snp/
