# Supplementary material for: Comprehensive genetic variant analysis reveals combination of KRAS and LRP1B as a predictive biomarker of response to immunotherapy in patients with non-small cell lung cancer
Source: J Exp Clin Cancer Res. 2025 Feb 27;44:75. doi: 10.1186/s13046-025-03342-6 (PMC11866712; doi:10.1186/s13046-025-03342-6)
Supplement: Supplementary file 4 — Supplementary Material 4: Additional file 4.pdf– Mutational signatures for LCG48: Profile of mutational signature analysis for patient LCG48. LCG48 was an outlier and presented a profile consisting of 92% SBS7a and SBS7b (cosine similarity of 0.984). Signatures associated with UV light exposure. Abbreviation used: SBS = Single base substitution. [file 13046_2025_3342_MOESM4_ESM.pdf]

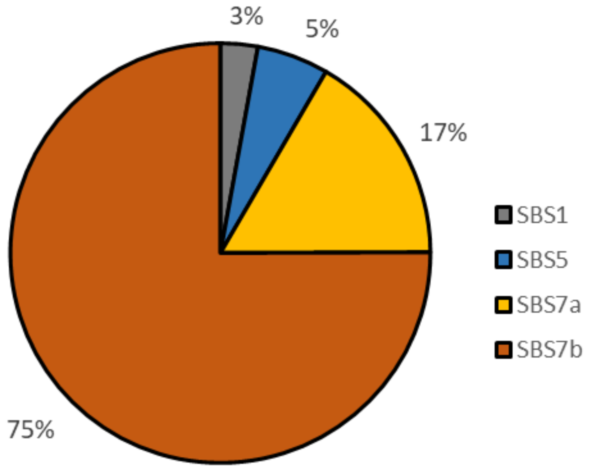


**Additional file 4. Mutational signatures for LCG48.** Profile of mutational signature analysis for patient LCG48. LCG48 was an outlier and presented a profile consisting of 92% SBS7a and SBS7b (cosine similarity of 0.984). Signatures associated with UV light exposure. Abbreviation used: SBS = Single base substitution.
