# Supplementary material for: Comprehensive genetic variant analysis reveals combination of KRAS and LRP1B as a predictive biomarker of response to immunotherapy in patients with non-small cell lung cancer
Source: J Exp Clin Cancer Res. 2025 Feb 27;44:75. doi: 10.1186/s13046-025-03342-6 (PMC11866712; doi:10.1186/s13046-025-03342-6)
Supplement: Supplementary file 10 — Supplementary Material 10: Additional file 10.pdf–CSMD3: Kaplan-Meier estimates comparing overall survival (A) and progression free survival (B) stratified on CSMD3 status. [file 13046_2025_3342_MOESM10_ESM.pdf]

Eklund et al. Additional file 10

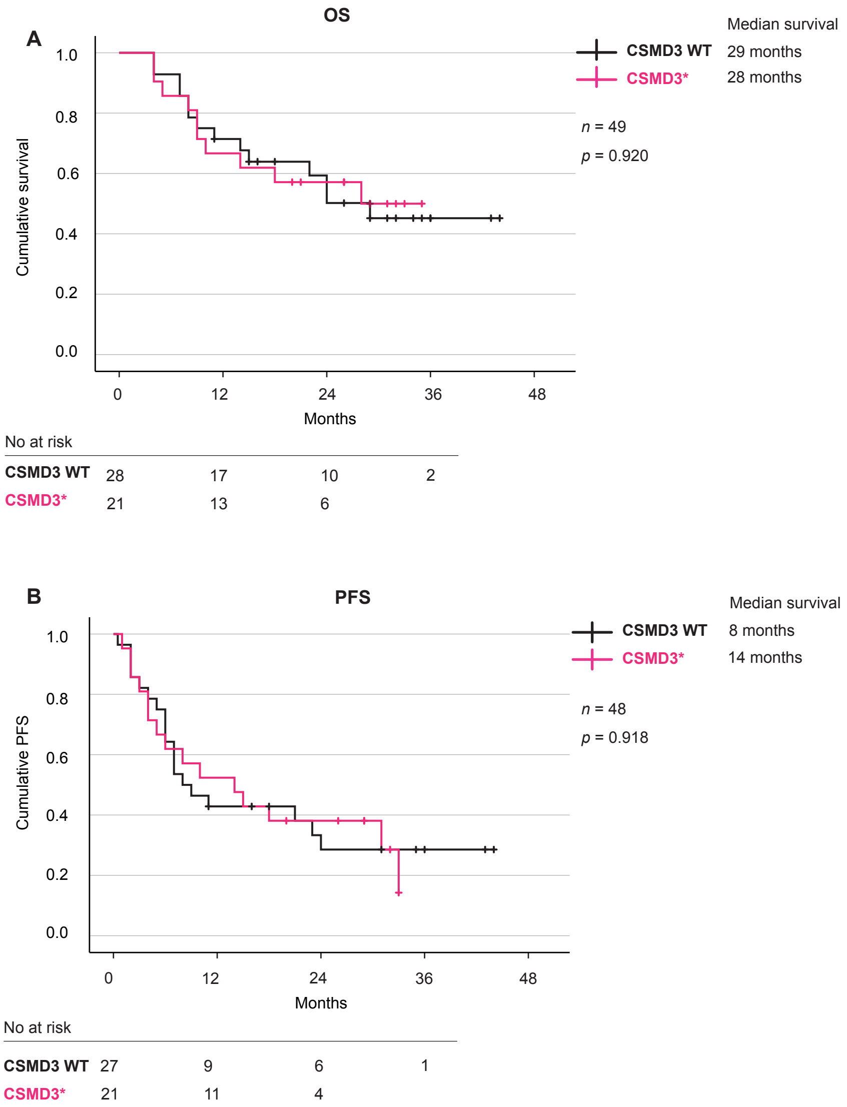

Additional file 10. CSMD3. Kaplan-Meier estimates comparing overall survival (A) and progression free survival (B) stratified on CSMD3 status.
