# Supplementary material for: Comprehensive genetic variant analysis reveals combination of KRAS and LRP1B as a predictive biomarker of response to immunotherapy in patients with non-small cell lung cancer
Source: J Exp Clin Cancer Res. 2025 Feb 27;44:75. doi: 10.1186/s13046-025-03342-6 (PMC11866712; doi:10.1186/s13046-025-03342-6)
Supplement: Supplementary file 11 — Supplementary Material 11: Additional file 11.pdf– Single and combined variants as predictive biomarkers: Kaplan-Meier estimates comparing overall survival (A) and progression free survival (B) and merged progression free survival (C) stratified on having neither, one of or combined KRAS* and LRP1B* variants. Kaplan-Meier estimates comparing merged progression free survival (D) for the combined KRAS* and LRP1B* variants population stratified on TP53* status. *DNA variant classified as P, LP, VUS++, VUS + and VUS. [file 13046_2025_3342_MOESM11_ESM.pdf]

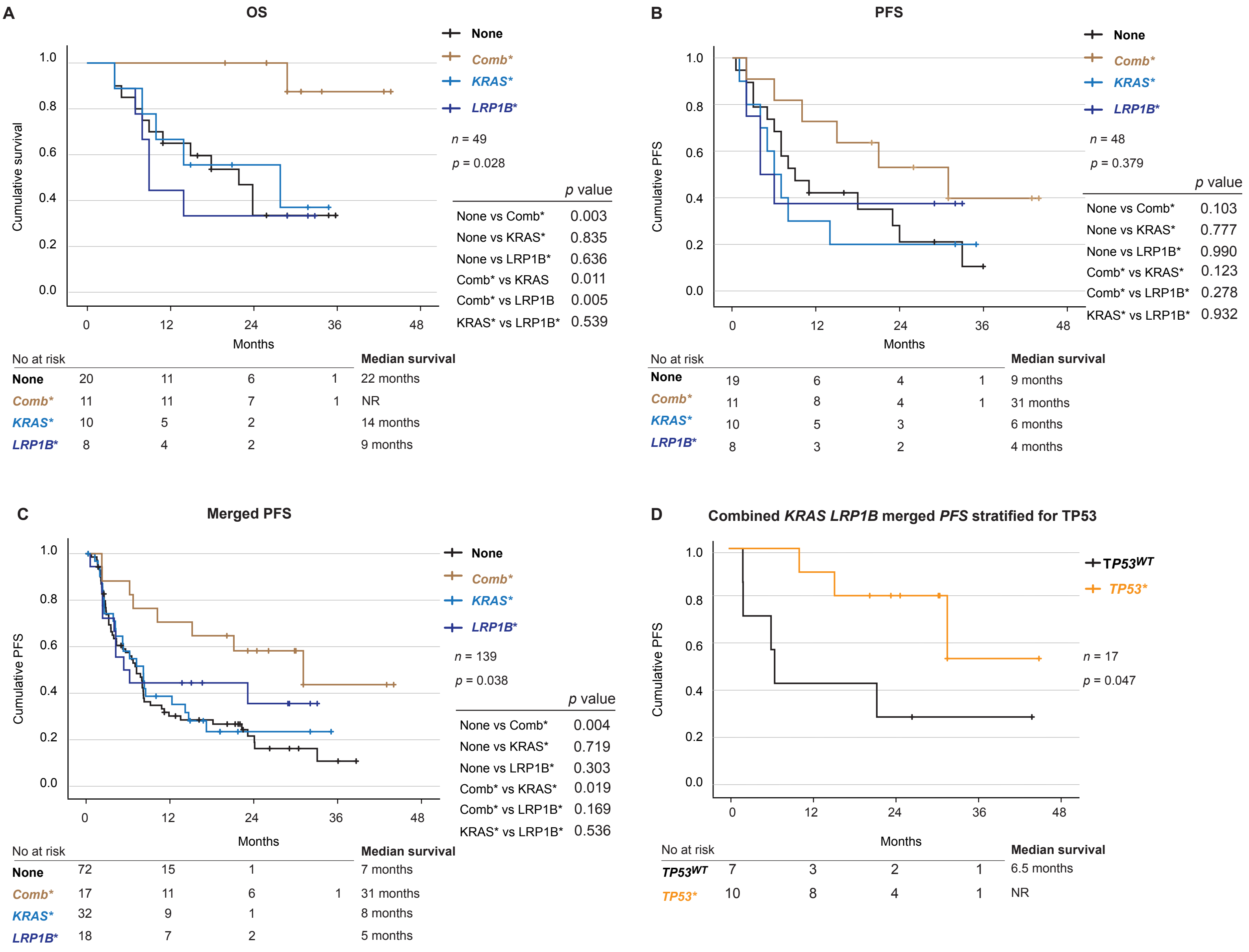

Additional file 11. Single and combined variants as predictive biomarkers. Kaplan-Meier estimates comparing overall survival (A) and progression free survival (B) and merged progression free survival (C) stratified on having neither, one of or combined KRAS* and LRP1B* variants. Kaplan-Meier estimates comparing merged progression free survival (D) for the combined KRAS* and LRP1B* variants population stratified on TP53* status.

*DNA variant classified as P, LP, VUS++, VUS+ and VUS
